# Comparable temperature-dependent predatory impacts of two invasive crayfish, *Pontastacus leptodactylus* and *Pacifastacus leniusculus*

**DOI:** 10.1007/s10530-026-03864-w

**Published:** 2026-07-03

**Authors:** James Hodson, Matthew Harwood, Kelvin Choy, Erin Cadley, Amarni-Jai Newman, Josie South

**Affiliations:** 1 South Aquatic Interactions Lab, School of Biology, Faculty of Biological Sciences, University of Leeds, Leeds, UK; 2 Water@Leeds, School of Biology, Faculty of Biological Sciences, University of Leeds, Leeds, UK

**Keywords:** Context-dependent impact, Functional response, Impact Assessment, Prey Response, Temperature-dependence

## Abstract

Narrow-clawed crayfish (*Pontastacus leptodactylus*) are an emergent invasive species in the UK and Europe, with limited data regarding their potential ecological impacts. Predicting ecological impact in current and future climate conditions is essential to inform policy and management actions. Therefore, we determined temperature-dependent predation of *P. leptodactylus* compared to the well-studied Signal crayfish (*Pacifastacus leniusculus*) on native prey *Gammarus pulex*, along a seasonal gradient (9℃, 12℃, 17℃, and 22℃) using functional response. *P. leniusculus* are wide-spread global invaders and are included as a well-known damaging invader reference species. Both species exerted strong predatory impact on *G. pulex* at all temperatures, however, when field abundance was considered *P. leptodactylus* have a higher relative impact driven by higher field abundance for our sampled populations. *P. leptodactylus* relative impact potential will increase with temperature up to ~ 17℃ and was exacerbated when prey (*G. pulex*) response to temperature change was incorporated, due to decreased prey abundance at higher temperatures. Our findings indicate *P. leptodactylus* overlooked potential as a damaging invader, both currently and in the future, and highlight the importance of considering resource and consumer response to changing climate in impact assessments. We recommend that stakeholders consider renewed management appraisals for populations of *P. leptodactylus* to reduce spread and population size to minimise ecological harm.

**Supplementary Information:**

The online version contains supplementary material available at 10.1007/s10530-026-03864-w.

## Introduction

Freshwaters are ecologically, economically and socially important, providing a wide array of ecosystem services and economic value to humans (Vári et al. 2022) and supporting a disproportionately high diversity for their area (Strayer and Dudgeon 2010); 9.5% of all animal species and 1% of vascular plants known worldwide (Balian et al. 2008). However, freshwater biodiversity is declining faster than terrestrial and marine realms (Darwall et al. 2018; Tickner et al. 2020) as a result of multiple threats including invasive non-native species, climate-change, over-exploitation, destruction/alteration of habitat and pollution (Strayer and Dudgeon 2010; Reid et al. 2019; Tickner et al. 2020). Invasive species are a major driver of biodiversity loss worldwide (Bellard et al. 2016; Doherty et al. 2016; Blackburn et al. 2019; IPBES 2019), particularly within freshwaters (Sala et al. 2000; Cox and Lima 2006) which are typically heavily invaded (Strayer 2010). This is due to the wide range of introduction sources: aquaculture, ornamental trade, and ballast water release, and relatively low barriers to dispersal within freshwaters (Padilla and Williams 2004; Moorhouse and Macdonald 2015; Faria et al. 2025).

Crayfish are particularly damaging freshwater invaders (Mathers et al. 2020b; O’Hea Miller et al. 2024), for example Signal crayfish (*Pontastacus leniusculus*) alter aquatic community structure through reduction of benthic fish, salmonid fry and macro-invertebrate abundance (Edmonds et al. 2011; Holdich et al. 2014; Galib 2020). This causes long term changes in macro-invertebrate community functional properties and composition (Mathers et al. 2020b). Frustratingly, invasive species impacts are typically observed after invasions are successful (Crooks 2005; Strayer et al. 2006), and eradication of established invasive crayfish remains challenging (Peay 2001; Gherardi et al. 2011; Simberloff 2021). Thus, to reduce future invasive impact, an emphasis should be on pre-emptively identifying potential new damaging invaders and implementing proactive policy to prevent invasions (Strayer 2010).

The Narrow-clawed crayfish (*Pontastacus leptodactylus*), native to the Ponto-Caspian region and widely introduced in Central Europe through the aquaculture pathway, is data deficient in terms of impact assessment compared to widely distributed invasive species like the Signal crayfish *P. leniusculus* or Red swamp crayfish *Procambarus clarkii* (Holdich et al. 2008; Twardochleb et al. 2013). Concerningly, non-native *P. leptodactylus* have been shown to have greater impact on gatherer and shredder invertebrates than *P. leniusculus* (Jackson et al. 2014). This could be exacerbated by climate change and associated temperature increases, which can increase abundance or feeding rates of invasive species (Cuthbert and Briski 2021; Teesalu et al. 2025). Furthermore, based on current *P. leptodactylus* distribution and predicted suitable invasion range there is potential for range expansion into northern Europe in 2050 (Hodson et al. 2024). Consequently, temperature-dependent impact assessments are essential to evidence gather for future management of this potential emergent threat (Bradley et al. 2023). These impact assessments must also consider temperature-dependent prey response, given asymmetry in predator and prey responses to temperature can alter impact (Öhlund et al. 2015; Pintanel et al. 2021; Meehan and Lindo 2023) for a more holistic, accurate impact assessment (Dickey et al. 2020; South et al. 2022).

Comparative Functional response (FR), which captures density dependent consumption by an invader and compares it to other species, is a commonly used impact assessment (Dick et al. 2017; Faria et al. 2025). It holds that, invasive species have greater per-capita consumptive effects on resources than natives, and that greater impact is associated with greater per-capita effects (Ricciardi et al. 2013; Dick et al. 2014). Attack rate and handling parameters derived from the FR can be used to calculate the Functional Response Ratio (FRR), a composite impact metric that is consistently higher in invaders than natives (Cuthbert et al. 2019). This can be combined with observed abundances of invaders in different contexts, to give a Relative Impact Potential (RIP), which provides a field contextualised impact assessment with greater predictive power (Laverty et al. 2017; Dickey et al. 2020). The RIP metric can also incorporate contextual prey abundance (RIPq) to explore whether resource response changes, offset or exacerbate ecological impact (South et al. 2022).

Thus, we used comparative FR experiments to calculate impact and relative impact potential of *P. leptodactylus* and *P. leniusculus* preying on *Gammarus* pulex across four temperatures (9°C, 12°C, 17°C and 22°C)*.* Comparison with *P. leniusculus* contextualises *P. leptodactylus* impact relative to a well-studied destructive invader. *Gammarus pulex* were used as prey as they are ubiquitous, omnivorous keystone species which alter nutrient cycling through leaf shredding and structuring of macro-invertebrate assemblages (Kelly et al. 2002; Navel et al. 2010). Experiments were carried out in the dark, to mimic natural crayfish feeding conditions, and reduce the chance of limited activity due to light exposure (Boyle et al. 2025). We predicted greatest impact (through Functional Response Ratio and Relative Impact Potential) of *P. leptodactylus* at 22°C, as this was the temperature closest to their reported thermal optima: 20–25℃ (Kir et al. 2025). We also predicted that incorporating prey response would exacerbate RIP at higher temperatures, as *G. pulex* are a cold adapted species (Foucreau et al. 2014).

## Methods

### Animal collection

Live *P. leptodactylus* were collected from Boshaw Whams Reservoir, West Yorkshire (N 53.547557°, W 1.77340°, May 2023), and *P. leniusculus* were collected from Settrington Beck, North Yorkshire (N 54.12001°, W 0.71992°; June, 2023) using trappy traps (Dimensions: 500 × 200 mm, diamond shaped mesh size: 30 × 20 mm) baited with cat or dog food. Traps were set overnight and retrieved in the morning (~ 18 h soak time). *Gammarus pulex* were collected using dip nets from Meanwood Beck, Leeds (N 53.83073°, S −1.576°) for use as prey. Boshaw Whams is a large unused reservoir stocked with rainbow trout, in West Yorkshire, Settrington Beck is a chalk stream tributary of the River Derwent in North Yorkshire.

### Animal maintenance

Crayfish were maintained in a controlled temperature laboratory in single sex, species specific holding tanks (internally 605 × 370 × 200 mm; Stocking Density: 10–16) in ~ 30 L of continuously aerated dechlorinated water held at 9.2 ± 1.0°C. Shelter was provided using PVC pipes, crayfish were fed ad libitum with dried sycamore leaves and wheatgerm pellets, and water was changed weekly to maintain water quality. *Gammarus pulex* were held in the same environmental conditions, in a clear plastic holding tank (450 × 300 × 300 mm) with glass beads as substrate and maintained on sycamore leaves.

### Experiments

Experimental arenas were plastic tanks (internally L605 × W370 × H200 mm) containing 24 L of continuously aerated dechlorinated water and no shelters, to avoid confounding FR results (per. South et al. 2019). Crayfish were randomly selected, sexed, patted dry and measured for mass (g) and carapace length (mm). Animals were size matched where possible, however, due to morphometric differences between species this was not always feasible, therefore a Kendall’s Tau correlation on mass and proportion was carried out within each species and temperature treatment, determining no significant correlations (Table SI1.2). No crayfish which had missing chelae or appendages, or which had recently moulted were used. Crayfish were then placed in experimental arenas without food for 24 h, standardising hunger levels and acclimating crayfish to the arenas. Prey (live *G. pulex*, total length: 0.5–1.5 mm) were supplied at six densities; 2, 6, 10, 20, 40 and 50 at four temperatures (n = 6 per density per temperature per species). Crayfish were allowed to feed for three hours in the dark before being removed and remaining live prey counted. Controls consisted of prey being supplied at a density of 50 without a crayfish at each temperature (n = 3 per temperature).

Crayfish were reused to limit animal use, however, were not used for the same treatment and not within 3 days of a previous experiment. Crayfish were marked to identify them with a small amount of non-toxic nail varnish which had no impact on behaviour.

### Temperature treatments

FR experiments detailed above were carried out at four temperatures in a controlled environment room: 9.2℃, 12℃, 17℃, and 22℃ ± 1.0℃. These temperatures were chosen to provide a temperature gradient reflective of seasonal temperature changes (Harwood et al. 2025) and to help predict changes in impact with predicted global temperature increases (Lowe et al., 2018). 22°C was included to provide a temperature closer to *P. leptodactylus* thermal optima and a ‘UK-extreme’ temperature. An incubator was used to acclimate the crayfish to the higher temperature at a rate of 1℃ per hour and then crayfish were acclimated at temperature for at least one week before experimentation.

### Statistical analysis

All analyses and modelling were carried out in R 4.2.1 (R Core Team, 2022).

#### Functional response

Functional response type was modelled using the R package ‘frair’ (v.0.5.100; Pritchard 2017) per Pritchard et al. (2017) (*frair::frair_test* and *frair_fit*). This uses a binomial logistic regression to determine if the proportion of prey consumed decreases with increasing density. A significantly negative first order term indicates a Type II functional response. Alternately, if a significantly positive first order term and significantly negative second order term is obtained it indicates a more sigmoidal Type III functional response (Juliano 1998). Functional responses were modelled using maximum likelihood estimation (MLE, v.1.0.25.1; Bolker 2023) and Rogers' (1972) Random Predator Equation to account for prey depletion:

1 $$N_{e} = N_{0} (1 - exp\left( {a\left( {N_{E} h - T} \right)} \right)$$

In which, *N*_*E*_ represents the number of prey eaten, *N*_*0*_ is the initial density of prey, *a* is the attack parameter, *h* is the handling parameter and *T* is the total time available.

Where categorical functional response models (Type II or III) could not be fitted (i.e. *P. leniusculus* at 9 °C), a flexible model was fitted (Real 1977; Vucic-Pestic et al. 2010b; Pritchard et al. 2017). This model extends Rogers’ random predator equation (Eq. 1) by incorporating a scaling exponent (*q*), bounded between 0 and 1, which allows the functional response to vary continuously between Type II (*q* = 0) and Type III (*q* = 1) forms (Eq. 2). Initial estimates of *q* were derived using two-parameter binomial logistic regression of the proportion of prey consumed against prey density. The first and second order terms from this allowed the curvature of the functional response to be quantified and used to inform model parameterisation. Based on these analyses, *q* was fixed at 0.2 in the flexible model, indicating a response that is closer to Type II but exhibits slight sigmoidal characteristics.

2 $$N_{e} = N_{0} \left( {1 - exp\left( {bN_{0}^{q} \left( {N_{E} h - T} \right)} \right)} \right)$$

In which, *N*_E_ is the number of prey consumed, *N*_0_ is the initial prey density, *b* is the search coefficient, *h* is handling time, *q* is the scaling coefficient and *T* is the total time available. Suitability of each non-categorical model was compared to its categorical analogue with Akaike information criterion (AIC).

Both categorical and flexible models were fitted to the data using the Lambert W function (Bolker 2008). For visual assessment the data were non-parametrically bootstrapped (*frair::frair_boot*; n = 2000) creating 95% confidence intervals around the mean functional response curve for each treatment. This allows phenomenological assessment of population level differences where overlapping confidence intervals indicates lack of statistical difference between functional response curves.

#### Functional response ratio

Data were also non-parametrically bootstrapped (*frair:: frair_boot*; n = 30) and had 95% outliers removed, to mechanistically compare the Functional Response Ratio (FRR) (*a/h* or for flexible models *b/h*) allowing for a composite comparison of both *a* and *h* (Cuthbert et al. 2019)*.* Data were over-dispersed so a generalised linear model (GLM) with a quasi-poisson error distribution was used to determine the effects of species and temperature on the FRR. The model was fitted with full interaction terms and simplified through step wise backward elimination. Model diagnostics and assumption checks were conducted using qq-plots and the breusch-pagan test for heteroskedasticity (*lmtest::bptest*; v0.9.40, Zeileis and Hothorn 2002). The function *car::Anova* (v3.11; Fox and Weisberg 2019) was used to interpret the model applying Type II (or Type III) Wald F-tests to assess the significance of predictors.

#### Relative Impact Potential (RIP)

FRR mean and SE generated from bootstrapping (frair:: frair_boot; n = 30) were also used in calculating RIP as in Dick et al., (2017) whereby:

3 $$RIP = \frac{FR X}{{FR Y}} \times \frac{AB X}{{AB Y}}$$

In which, *FR* refers to functional response or an equivalent impact metric, and *AB* refers to numerical response. *FR X* and *AB X* refer to the impact and abundance of population X, *FR Y* and *AB Y* refer to the same for population Y. In our case FR was the FRR; attack rate/handling time. Catch per unit effort (CPUE) for each species, the mean number of individuals caught per trap per night, was used for the numerical response as a proxy for the abundance of adult crayfish. CPUE was based on catch data of the sampled *P. leptodactylus* (Harwood et al. 2025) and *P. leniusculus* populations across different temperatures (Harwood and Hodson, Unpublished), and temperature treatments were matched to the closest temperature with recorded CPUE (SI2). Whilst baited traps used to catch crayfish can be size-biased to larger crayfish this should not affect relative abundance differences between temperatures. RIP was calculated for intraspecific comparisons between temperature treatments, and inter-species comparison within temperature treatments. Each species-temperature treatment combination RIP was visualised using a biplot, allowing for assessment of all combinations impact relative to each other. *Pacifastacus leniusculus* at 9℃ were not included in RIP analysis due to non-significant handling times which produce unreliable maximum feeding estimates and FRRs.

#### RIPq

RIP*q* was calculated as in South et al., (2022) accounting for prey abundance changes with temperature using a resource reproductive qualifier (RRQ) based on the average number of eggs per ovigerous females at different temperatures as observed by Hynes (1955), whereby:

4 $$RRQ = 1/\frac{Reproductive\,Output\,at\,higher\,temperature}{{Reproductive\,Output \,at\,lower\,temperature}}$$

5 $$RIPq = RIP \times RRQ$$

Temperatures observed by Hynes (1955) did not exactly match our temperature treatments. Where there was no exact match e.g. no observation of egg numbers at 12℃ to match our 12℃ condition, an average of egg numbers was taken between the closest relevant temperatures observed by Hynes. The highest temperature observed by Hynes (1955) was 15.3℃, so the egg numbers observed here were used for both 17 and 22℃ (SI3).

Differences in RIP and RIPq were visualised across temperature comparisons of 12 to 9℃, 17 to 12℃, and 17 to 9℃. RIPq was also generated and visualised for *Gammarus fossarum* using a different measure of reproductive output i.e. the fecundity index (Number of eggs/wet weight of female) provided by (Pöckl 1993) to test broader applications (Figure SI4a).

## Results

Control prey survival was > 99% so prey mortality in the experimental groups was attributed to predation.

### Functional response types

A significant Type II response, typical of impactful invasive species, was found for all treatments apart from *P. leniusculus* at 9℃ (Table 1). For *P. leniusculus* at 9℃, diagnostic two-term logistic regressions indicated that categorical Type III functional response models were inappropriate for this treatment (Table 1). Thus, it was modelled as a flexible type with the scaling exponent (*q*) fixed at 0.2, indicating a response between Type II and III.

**Table 1:** Functional mechanistic parameters (a, b, h, and 1 h) associated significance levels using Rogers’ random predator equation, bias accelerated and corrected 95% confidence intervals (BCa CIs) for a, b and h, scaling co-efficient (q), and the functional response ratio (a/h) for *Pontastacus leptodactylus* and *Pacifastacus leniusculus* under different temperature conditions

Species	Temperature (℃)	First order term (*p-*value)	FR Type	*a* (*p*-value)	*a* 95% BCa CI	*h* (*p*-value)	*h* 95% BCa CI	*b* (*p*-value)	*B* 95% BCa CI	*q* (*p*-value)	Maximum feeding estimate/3 h (1/*h*)	Functional response ratio (*a*/*h* or *b*/*h*)
*P. leptodactylus*	9	−0.02 (< 0.0001)	II	1.11 (< 0.0001)	0.22–0.61	0.023 (< 0.0001)	0–0.15	–	–	–	43.47	48.26
*P. leptodactylus*	12	−0.03 (< 0.0001)	II	1.93 (< 0.0001)	1.4–2.8	0.022 (< 0.0001)	0.005–0.04	–	–	–	45.45	87.73
*P. leptodactylus*	17	−0.01 (< 0.0001)	II	1.17 (< 0.0001)	0.8–1.62	0.015 (< 0.0001)	0–0.033	–	–	–	66.66	78
*P. leptodactylus*	22	−0.05 (< 0.0001)	II	2.28 (< 0.0001)	1.02–4.19	0.034 (< 0.0001)	0.012–0.073	–	–	–	29	67.06
*P. leniusculus*	9	−0.07 (0.0026)	Non-categorical	–	–	0.000083 (0.99)	−0.39–0.40	0.84 (0.0023)	0.28–2.67	0.030 (0.84)	12,048.19	10,120.48
*P. leniusculus*	12	−0.12 (< 0.0001)	II	4.88 (< 0.0001)	3.24–8.26	0.033 (< 0.0001)	0.019–0.048	–	–	–	30	147.88
*P. leniusculus*	17	−0.03 (< 0.0001)	II	2.24 (< 0.0001)	1.32–3.53	0.020 (< 0.0001)	0.004–0.034	–	–	–	50	112
*P. leniusculus*	22	−0.11 (< 0.0001)	II	5.29 (< 0.0001)	3.22–9.65	0.037 (< 0.0001)	0.022–0.055	–	–	–	27.03	142.97

### FR curves under different temperature

#### Between species

**Fig. 1:**
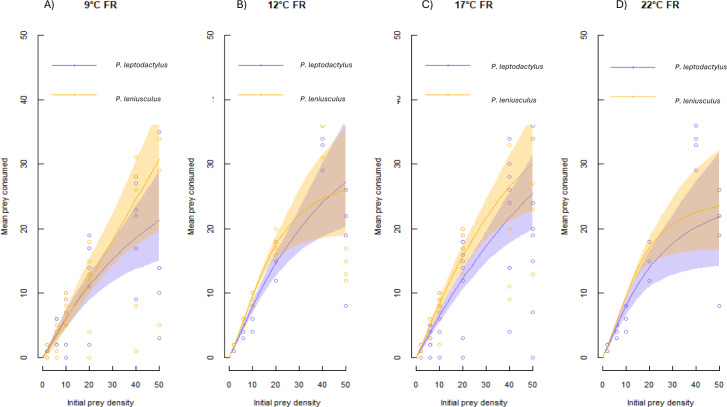
Comparison of species functional responses curves, of *Pontastacus leptodactylus* and *Pacifastacus leniusculus* towards *Gammarus pulex* split by temperature treatments: **A** 9℃, **B** 12℃, **C** 17℃, **D** 22℃. Shaded areas are 95% bootstrapped CIs, and darker areas represent overlap. Coloured points represent raw data of prey consumed

**Fig. 2:**
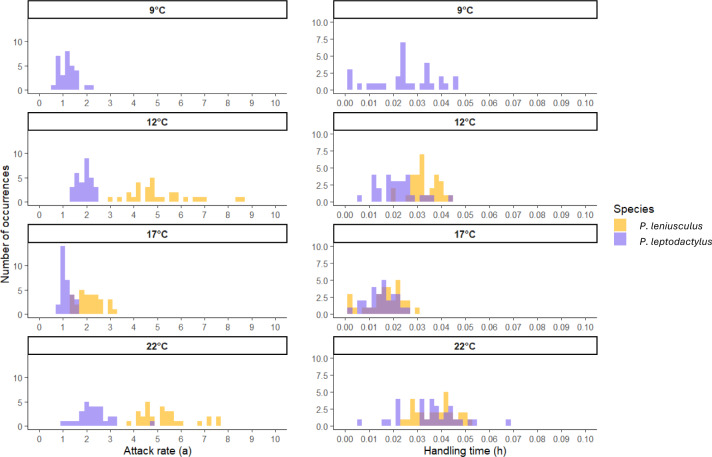
Variation in bootstrapped estimates (*n* = 30) of attack rates and handling times for functional responses of narrow-clawed crayfish (*Pontastacus leptodactylus*) and signal crayfish (*Pacifastacus leniusculus*) across species and temperature treatment

At 12°C and 17°C *P. leniusculus* functional response differed from *P. leptodactylus* curves, possessing significantly different curves at low densities (ca. densities 0–15, and 15–20 respectively) driven by a higher attack parameter, indicated by curve steepness (Table 1; Fig. 1B and C). At 9 and 22℃ *P. leniusculus* and *P. leptodactylus* had comparable functional responses indicated by overlapping confidence intervals (Table 1; Fig. 1A and D). For *P. leniusculus* at 9℃ the FR curve does not plateau as foraging saturation did not occur, producing an unreliable and non-significant handling time. Consequently, the resulting maximum feeding estimate and functional response ratio (FRR) are not derived for *P. leniusculus* at 9℃. *P. leptodactylus* had similar if not slightly faster handling time than *P. leniusculus* but conversely slower attack rates (Table 1, Fig. 2). Specifically, *P. leptodactylus* attacked prey less frequently than *P. leptodactylus*, however once prey were caught they were eaten slightly faster.

#### Within species

Functional response curves had broadly overlapped confidence intervals for *P. leptodactylus* compared between temperature across all densities, whereas for *P. leniusculus* curves broadly overlapped at prey densities above 20 across all temperatures (SI5). Thus, functional response was broadly similar across all temperatures in both species respectively.

### FRR

#### Between species

**Fig. 3:**
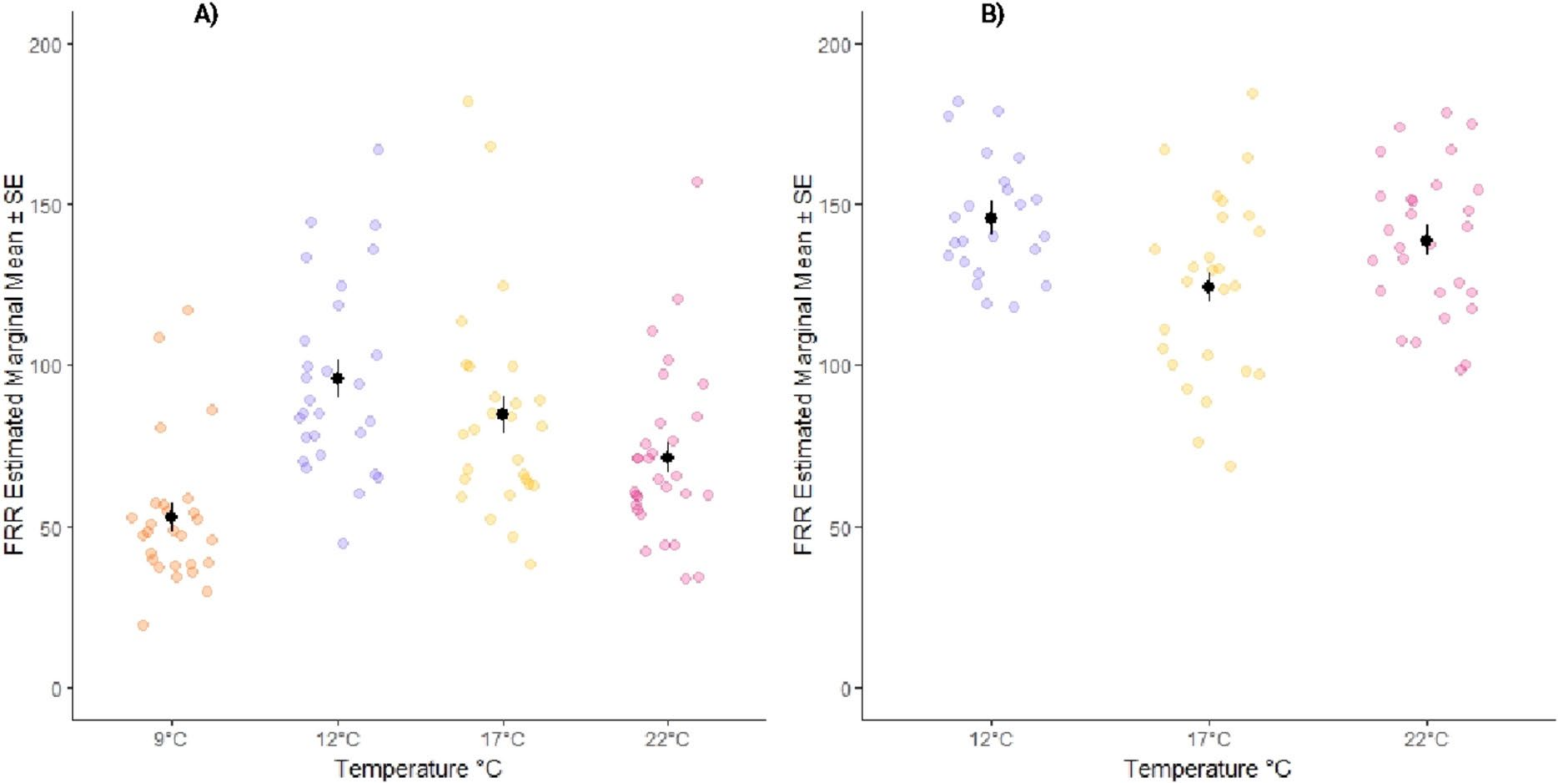
**A** FRR estimated marginal means at 4 temperatures for *Pontastacus leptodactylus*. **B** FRR estimated marginal means at 3 temperatures for *Pacifastacus leniusculus*. Black dots and bars represent mean ± SE, coloured dots represent raw data comparisons)

The interaction between temperature and species significantly affected FRR values (Table 2). As temperatures increased from 12 to 17℃ to 22℃ in *P. leptodactylus* FRR decreases, whereas for *P. leniusculus* FRR dips at 17℃, but remains relatively similar at 12℃ and 22℃ (Fig. 3). Across all temperatures, *P. leptodactylus* had a smaller per-capita impact than *P. leniusculus* indicated by a significantly lower FRR (*z* = −11.646, *p* =  < 0.0001). Please see SI6 for mechanistic comparisons of attack rate and handling times.

**Table 2:** Model terms for all variables from a generalised linear model with a quasi-poisson error distribution used to determine differences in FRR based on species (*Pontastacus leptodactylus* or *Pacifastacus leniusculus*), and temperature (12℃, 17℃ and 22℃ interactions, using a Type 3 ANOVA and χ2 to report the effects. 9℃ is not included, as FRR was only available for *P. leptodactylus*

Model terms	*X*^*2*^	*df*	*p*-value
Species	34.085	1	< 0.0001
Temperature	17.339	2	0.0001
Species*Temperature	18.455	2	< 0.0001

#### Within species

Temperature altered *P. leptodactylus* impact (FRR; *x*^*2*^ = 51.655, *df* = 3* p* =  < 0.0001), impact was significantly lower at 9℃ compared to the other temperatures but there were no differences between impacts at 12–17℃ and 17–22℃ (Fig. 3A, Table 3). Temperature also significantly impacted *P. leniusculus* impact (FRR; *x*^*2*^ = 22.016, *df* = 2* p* =  < 0.0001), which was significantly higher at 12 or 22°C respectively compared to 17°C (Fig. 3B, Table 3). Please see SI6 for mechanistic comparisons of attack rate and handling times.

**Table 3:** Pairwise comparison results from type II sum of squares used to determine significant differences in FRR for *Pontastacus leptodactylus* and *Pacifastacus leniusculus* within species at different temperature conditions: 9℃, 12℃, 17℃ and 22℃, 9℃ was not included in comparison for *P. leniusculus* due to non-significant handling times

Species	Comparison	*z*	*p*-value
*P. leptodactylus*	9–12℃	−5.68	< 0.0001
*P. leptodactylus*	9–17℃	−4.376	< 0.0001
*P. leptodactylus*	9–22℃	−2.796	0.0038
*P. leptodactylus*	12–17℃	1.353	0.42
*P. leptodactylus*	12–22℃	3.133	0.0021
*P. leptodactylus*	17–22℃	1.73	0.19
*P. leniusculus*	12–17℃	3.11	0.0002
*P. leniusculus*	17–22℃	−2.23	0.0001
*P. leniusculus*	12–22℃	0.99	0.984

### Relative Impact Potential (RIP)

#### FRR RIP

**Fig. 4:**
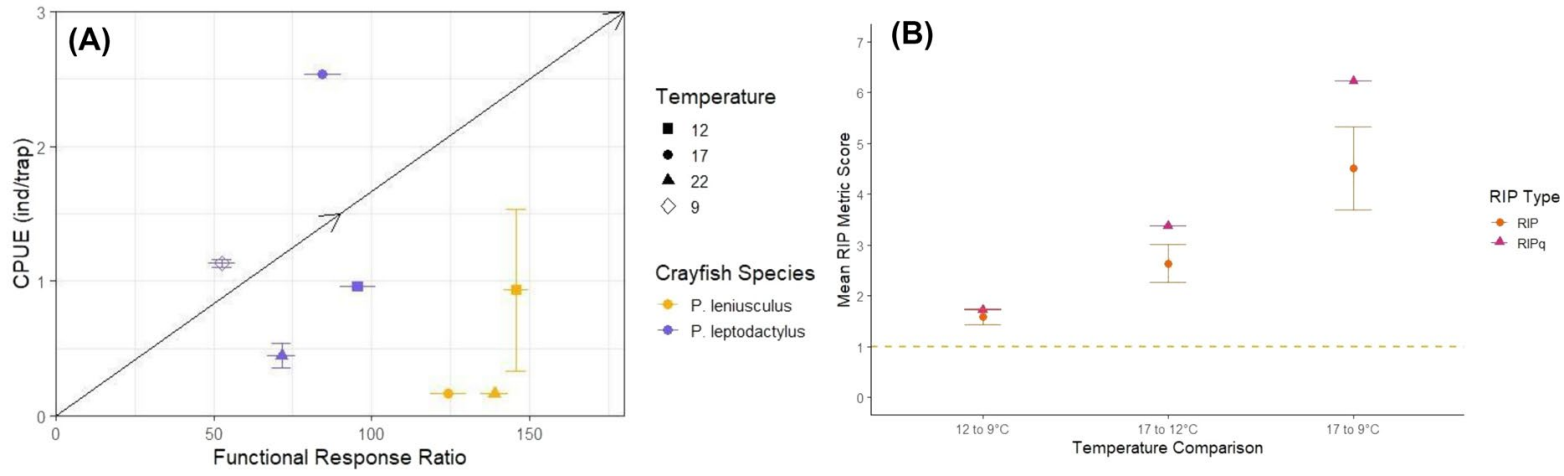
**A** RIP biplots using catch per unit effort (CPUE) as abundance proxy and functional response ratio for *Pacifastacus leniusculus* and *Pontastacus leptodactylus* as impact metric. Ecological impact increases from bottom left to top right. **B** Relative Impact Scores (RIP; based on Functional Response Ratio) and RIPq scores which incorporate the ‘resource reproductive qualifier’ for between-temperature comparisons for *Pontastacus leptodactylus*. The resource reproductive qualifier was based on average egg number per ovigerous female *Gammarus pulex* at different temperatures. Points above the dashed yellow line indicate greater Relative Impact Potential from the first temperature stated compared to the second e.g. at 12℃ compared to 9℃

Using the Functional Response Ratio (FRR) for RIP the highest relative impact on *G. pulex* is at 17℃ for *P. leptodactylus* and 12℃ for *P. leniusculus*. This is driven by high abundance (CPUE) for *P. leptodactylus* at 17 ℃ and high impact (FRR) for *P. leniusculus* at 12℃ (Fig. 4A). *P. leptodactylus* at 9℃ have the lowest relative impact driven by the lowest impact and a low abundance (Table SI4 for RIP values).

#### RIPq

Incorporating the resource reproductive qualifier (prey abundance) increased the relative impact potential at 17℃ across all temperature comparisons except from 22 to 17℃ where the RRQ was 1 because the same average egg number per females was used (SI3, Fig. 4B, Figure SI4b for all temperatures).

## Discussion

We combined FR experiments to compare the predatory impact of both *Pontastacus leptodactylus* and *Pacifastacus leniusculus* under four different temperature conditions: 9, 12, 17 and 22℃. *P. leptodactylus* and *P. leniusculus* both show similar Type II functional response across all temperatures, excepting *P. leniusculus* at 9℃. *P. leptodactylus* and *P. leniusculus* *per-capita* impact, based on FRR, varied significantly with temperature and was greatest at 12℃, contrary to our predictions. Across both species, *P. leptodactylus* had the greatest relative impact potential at 17℃. This was exacerbated when prey response to temperature change was incorporated.

Impact was comparable between *P. leptodactylus* and *P. leniusculus* at all temperatures as indicated by broadly overlapping Type II FR curves (Bollache et al. 2007; Alexander et al. 2014). Furthermore, at 12 and 17℃ FRR values of *P. leptodactylus* are similar or greater than mean FRR benchmark values for invaders with known high ecological impact (83.36) reported by (Cuthbert et al. 2019). Consequently, *P. leptodactylus* will likely be an impactful invader, particularly within lentic European freshwaters, over-predating *G. pulex* and destabilising broader freshwater systems and communities in a similar fashion to *P. leniusculus* (Dunoyer et al. 2014; Galib 2020; Mathers et al. 2020b). Whilst our study was simplistic and based on arenas without structural complexity which can tend to Type II FR’s (Vucic-Pestic et al. 2010a), we consider impact relative to a known damaging invader thus similar results can be viewed as a proxy for high impact. Ultimately, significantly lower FRR in *P. leptodactylus* compared to *P. leniusculus* does suggest that per capita, *P. leptodactylus* would have lower predatory impacts on *G. pulex* than *P. leniusculus*. This is driven by *P. leniusculus* attack rate being almost twice as high as *P. leptodactylus* in all temperature conditions. Future research would benefit from including higher prey densities, to allow feeding rates at all temperatures (i.e. 9℃) to reach a saturation plateau and subsequent calculation and comparison of FRR metrics across a wider thermal range.

When incorporating field abundance for our sampled populations, *P. leptodactylus* have a greater relative impact potential than *P. leniusculus* due to higher abundance. *P. leptodactylus*’ higher abundance is surprising given *P. leniusculus* are typically more fecund (Chybowski 2013; Cìlbìz 2020) but likely reflects that *P. leptodactylus* were only sampled from lentic environments in which they had high abundance (Harwood et al. 2025), and *P. leniusculus* from lotic, which may drive the abundance difference. For example, *P. leniusculus* are less likely to establish in high-flow lotic environments, which may reduce our sampled populations abundance (Mathers et al. 2020a). This emphasises how contexts like hydrological regime influence impact and must be considered when making impact predictions, particularly when transferring knowledge across species or locations. It demonstrates how in these conditions, narrow-clawed crayfish have a comparatively greater RIP however these findings should be generalised cautiously, given they apply to the specific localities sampled. In other population comparisons we may see a different trend. It is also important to note that these abundance estimates were based on CPUE which, driven by life history or climatic variables other than temperature, can vary substantially seasonally (Richards et al. 1996; Zimmerman and Palo 2012; Harwood et al. 2025) which could bias our impact estimates. However, given true abundance is logistically hard to acquire and temperature ultimately does still influence CPUE (Araujo and Romaire 1989; Richards et al. 1996), we believe that CPUE functions well as a proxy of temperature-dependent relative abundance.

For *P. leptodactylus*, our results provide evidence of a population specific thermal optima of ~ 12–17℃; lower than the optima of 20–25℃ observed in Turkey (Kir et al. 2025). This adds to the body of evidence for observations of population specific functional responses for freshwater invaders like *Faxonius limosus*, *Faxonius rusticus* and *Pseudorasbora parva* (Boets et al. 2019; Grimm et al. 2020; Chicatun et al. 2024) and suggests, if possible, impact assessments be carried out at the population not species level. Our lower optima likely reflect temperatures at Boshaw Whams reservoir, Yorkshire, which ranged from 0.7–19℃ (Harwood et al. 2025). The greater impact at 12–17℃ is consistent with previous research finding highest FRs at intermediate temperatures (Uiterwaal and DeLong 2020). Consequently, large temperature increases above 17℃ may reduce the ecological impact of *P. leptodactylus* within West Yorkshire particularly given gamete production is impaired from 19℃ which may simultaneously reduce abundance (Farhadi and Harlıoglu 2018).

As well as consumer response to different contexts like temperature, prey response should also be incorporated into impact assessments for more accurate impact predictions (Kumschick et al. 2015; Urban et al. 2016; South et al. 2022). Ideally this would be based on empirically derived relationships between temperature (or another moderator) and abundance, unaffected by other factors like seasonal life history traits. However, as in our case, these are not always available and field abundances and temperatures provide a valid alternative. British or cold-adapted *Gammarus pulex* populations show reduced reproductive functioning from 10–20℃, and reductions in survival from 15–30℃ (Welton and Clarke 1980; Foucreau et al. 2014). Coupled with the high thermal tolerance of *P. leptodactylus* (Kir et al. 2025) this suggests a predator–prey asymmetry in thermal responses, which may alter impact (Öhlund et al. 2015; Pintanel et al. 2021; Meehan and Lindo 2023). Considering consumer and prey abundance our RIPq results reflect that reduced reproductive output of *G. pulex* at higher temperatures will exacerbate *P. leptodactylus* impact. Thermal asymmetry can also occur in differing levels of fitness, metabolism, growth rates and drive observed patterns. For example, as temperatures increases from 5–15 ℃, *G. pulex* show an increase in motility (Vellinger et al. 2012). The significant decrease in FRR, from 12–17℃ for *P. leniusculus*, is driven by reduced attack rate. Perhaps increased motility of *G. pulex* at temperatures close to 15 ℃ may reduce opportunity to attack or successful attacks, which are computed by FR analyses as the same entity, producing the observed reduction in impact. Consequently, for accurate impact assessments, it is imperative we consider a variety of prey responses to the observed context alongside predator response.

While *Pacifastacus lenuisculus* is considered to be a pervasive threat to native species through predation and pathogen transfer, *Pontastacus leptodactylus* has been overlooked for management interventions. Nonetheless, *P. leptodactylus* is predicted to be an ecologically damaging invasive species now, and in the near future, whose relative impact potential due to predation will increase with temperature up to ~ 17℃. When incorporating prey response to temperature, into our predictions, we find an even greater relative impact, emphasising the threat of *P. leptodactylus* and highlighting the importance of including prey-response in impact assessments. Additionally, *P. leptodactylus* potential suitable range, currently and in the future, gives room for expansion from the current invasive distribution (Hodson et al. 2024). Therefore, we recommend that stakeholders consider renewed management appraisals for populations of *P. leptodactylus* to reduce spread and population size to minimise ecological harm.

## Supplementary Information

Below is the link to the electronic supplementary material.

Supplementary file1 (DOCX 3551 KB)

Supplementary file2 (XLSX 19 KB)

## Data Availability

Functional Response Data is available on Figshare here 10.6084/m9.figshare.32429145.
