# Supplementary material for: Comparable temperature-dependent predatory impacts of two invasive crayfish, Pontastacus leptodactylus and Pacifastacus leniusculus
Source: Biol Invasions. 2026 Jul 3;28(7):161. doi: 10.1007/s10530-026-03864-w (PMC13328125; doi:10.1007/s10530-026-03864-w)
Supplement: Supplementary file 1 — Supplementary file1 (DOCX 3551 KB) [file 10530_2026_3864_MOESM1_ESM.docx]

**Supplementary Information**

Contents

[Supplementary Information 1 1](#_heading=h.ch532h4sljnr)

[Supplementary Information 2 4](#_heading=h.mb5vm3aizhlw)

[Supplementary Information 3 4](#_heading=h.1e3kz0id27fv)

[Supplementary Information 4 5](#_heading=h.46cmpxdvzh34)

[Supplementary Information 5 7](#_heading=h.3lbt398wveos)

[Supplementary Information 6 8](#_heading=h.pr7ctkdian8w)

[Bibliography 9](#_heading=h.s0bayb8aezl8)

## Supplementary Information 1

**SI1.1:** Mass and carapace length descriptives for species-temperature treatments

| **Treatment** | **Sex ratio (Male/Female)** | **Mean ± SE** | **Range (min-max) (mm)** |
| --- | --- | --- | --- |
| **Mass (g)** |  |  |  |
| *P. leptodactylus* (9℃) (n=36) | 18/18 | 72.99 ± 7.08 | 174.6 (22.6 – 197.2) |
| *P. leptodactylus* (12℃) (n=26) | 14/12 | 48.37 ± 5.09 | 101.6 (13.0-114.6) |
| *P. leptodactylus* (17℃) (n=62) | 31/31 | 60.18 ± 3.95 | 185 (17.5 – 203.2) |
| *P. leptodactylus* (22℃) (n=24) | 10/14 | 60.6 ± 4.81 | 98 (31.4-129.4) |
| *P. leniusculus* (9℃) (n=36) | 20/16 | 38 ± 4.02 | 72.8 (16.3 – 89.1) |
| *P. leniusculus* (12℃) (n=24) | 12/12 | 48.37 ± 3.86 | 67.5 (18.0 – 85.5) |
| *P. leniusculus* (17℃) (n=36) | 15/21 | 31.61 ± 2.66 | 70.2 (15.1 – 85.3) |
| *P. leniusculus* (22℃) (n=24) | 10/14 | 32.9 ± 3.6 | 69.1 (15.1 – 84.2) |
| **Carapace Length (mm)** |  |  |  |
| *P. leptodactylus* (9℃) (n=36) | 18/18 | 65.61 ± 2.03 | 46.4 (46.5 – 92.9) |
| *P. leptodactylus* (12℃) (n=26) | 14/12 | 54.47 ± 2.36 | 40.4 (36.2 – 76.6) |
| *P. leptodactylus* (17℃) (n=62) | 31/31 | 61.41 ± 1.41 | 52.2 (41.3 – 93.5) |
| *P. leptodactylus* (22℃) (n=24) | 10/14 | 65.1 ± 1.62 | 38.57 (52.6 – 91.3) |
| *P. leniusculus* (9℃) (n=36) | 20/16 | 47.38 ± 1.56 | 38.5 (36 – 74.5) |
| *P. leniusculus* (12℃) (n=24) | 12/12 | 45.48 ± 1.27 | 25.8 (34.0 – 59.8) |
| *P. leniusculus* (17℃) (n=36) | 15/21 | 44.89 ± 1.05 | 27.6 (34.2 – 61.8) |
| *P. leniusculus* (22℃) (n=24) | 10/14 | 46.85 ± 1.23 | 21.8 (38.4 – 60.2) |

**Table SI1.2:** Kendall’s correlation between proportion of prey consumed and mass split by temperature and crayfish species. Kendall’s Tau is provided to 3 s.f. *p*-values provided by *p*-value approximation due to ties in the ranking.

| **Species** | **Temperature** | **Kendall’s T** | ***p*-value** |
| --- | --- | --- | --- |
| *Pontastacus leptodactylus* | 9℃ | -0.0960 | 0.419 |
| *Pontastacus leptodactylus* | 12℃ | 0.0254 | 0.859 |
| *Pontastacus leptodactylus* | 17℃ | -0.169 | 0.058 |
| *Pontastacus leptodactylus* | 22℃ | -0.126 | 0.404 |
| *Pacifastacus leniusculus* | 9℃ | -0.140 | 0.239 |
| *Pacifastacus leniusculus* | 12℃ | -0.0321 | 0.835 |
| *Pacifastacus leniusculus* | 17℃ | -0.0524 | 0.666 |
| *Pacifastacus leniusculus* | 22℃ | -0.0804 | 0.604 |


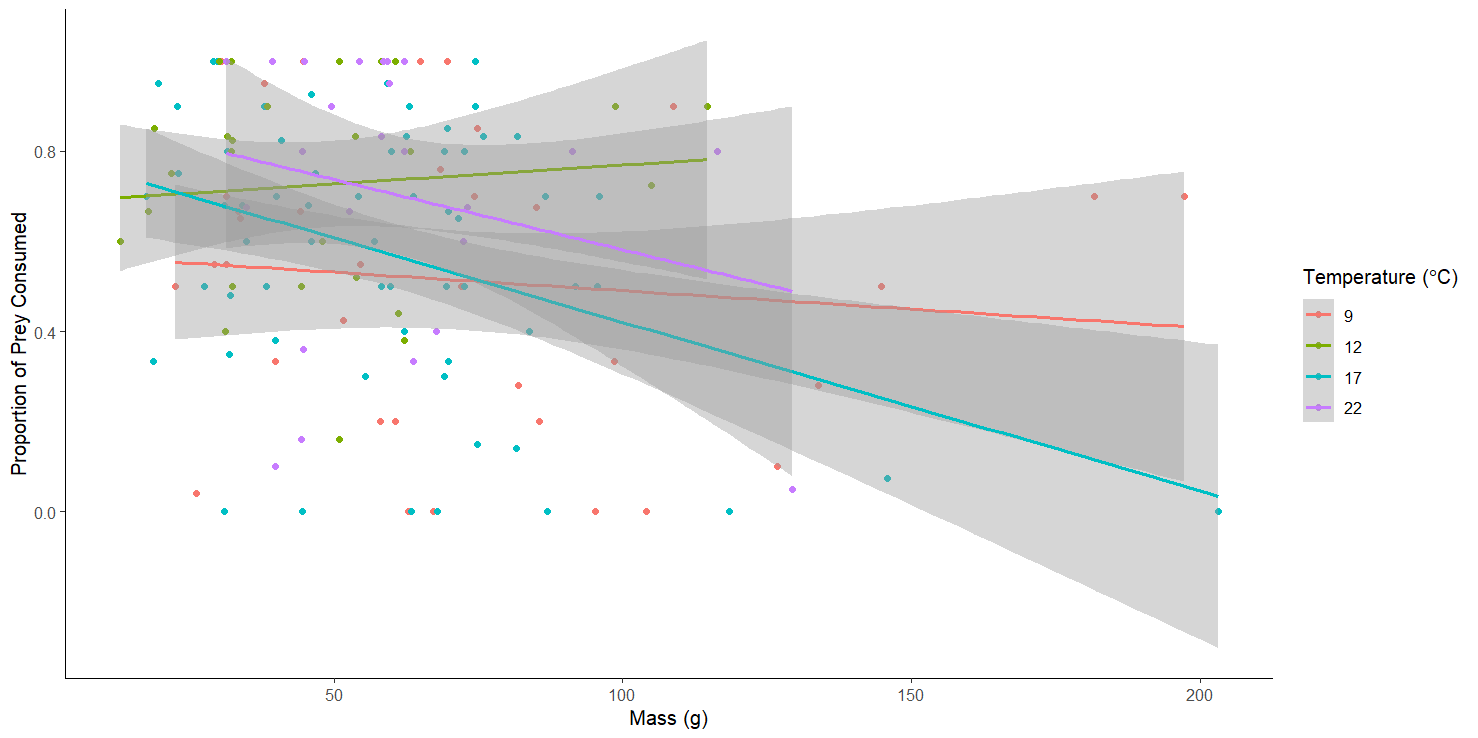


**Figure SI1.1:** Interaction between mass and temperature on the proportion of prey consumed for *Pontastacus leptodactylus*.

**
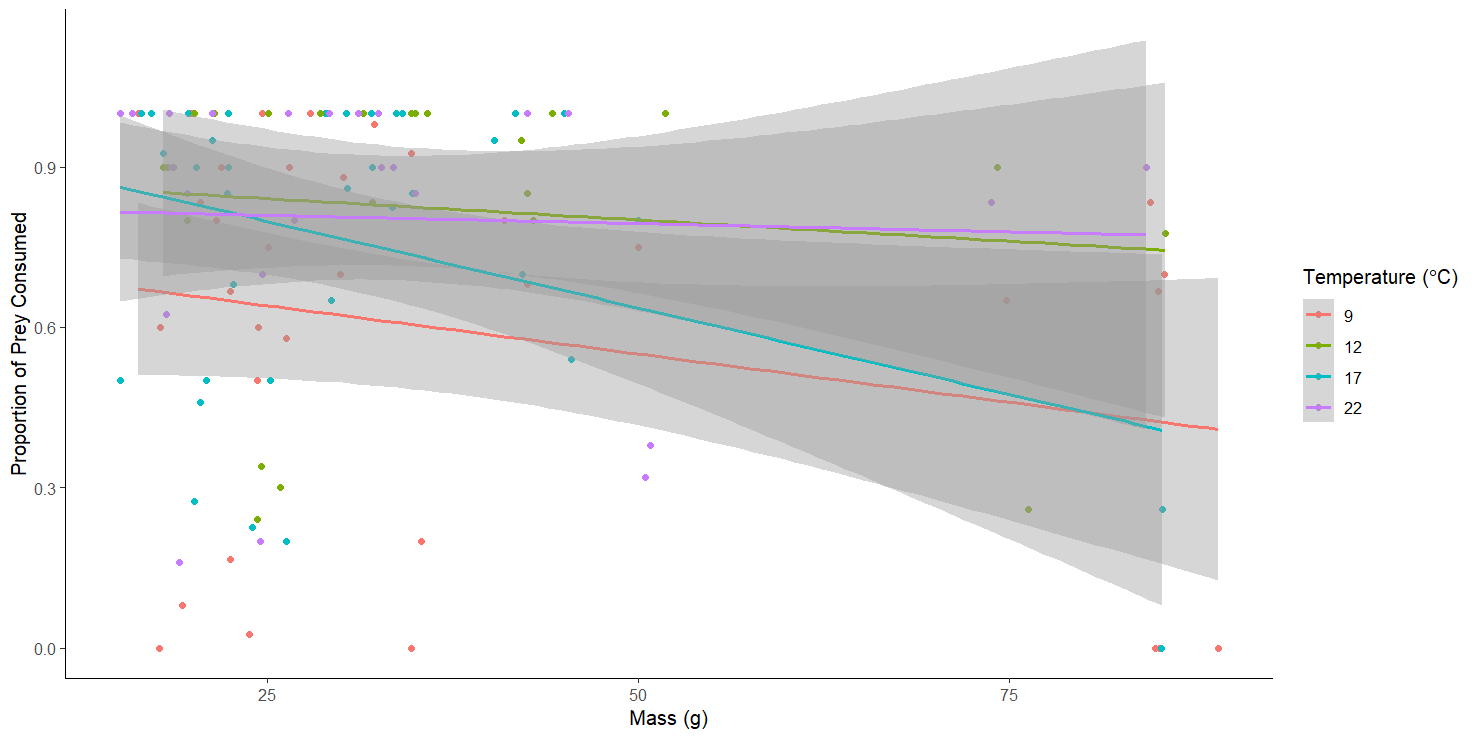
**

**Figure SI 1.2:** Interaction between mass and temperature on the proportion of prey consumed for *Pacifastacus leniusculus*.

## Supplementary Information 2

See “Supplementary Material 2 – Crayfish CPUEs.xlsx” for temperature matched crayfish CPUEs.

## Supplementary Information 3

**SI3:** Average numbers of eggs per ovigerous females used for RIPq calculations taken from Table 1 of Hynes (1955) matched to temperature conditions

| **RIPq temperature condition** | **Temperatures in Hynes (1955)** | **Number of Eggs** |
| --- | --- | --- |
| 9°C | 8.2 and 9.8°C (average of egg numbers at these temperatures) | 18.8 |
| 12°C | 11.5°C | 17.4 |
| 17°C | 15.3°C | 13.6 |
| 22°C | 15.3°C | 13.6 |

## Supplementary Information 4

**Tables SI 4:** Mean RIP values from interspecies across the same temperature, and intraspecies comparisons across different temperatures. Species are *Pontastacus leptodactylus* and *Pacifastacus leniusculus*. RIP values to 3 s.f., values above 1 indicate greater relative impact.

| **Species** | **Comparison** | **RIP value** |
| --- | --- | --- |
| *P. leptodactylus* | 12℃ - 9℃ | 1.593 |
| *P. leptodactylus* | 17℃ - 9℃ | 4.50 |
| *P. leptodactylus* | 22℃ - 9℃ | 0.515 |
| *P. leptodactylus* | 17℃ - 12℃ | 2.63 |
| *P. leptodactylus* | 22℃ - 12℃ | 0.321 |
| *P. leptodactylus* | 22℃ - 17℃ | 0.136 |
| *P. leniusculus* | 17℃ - 12℃ | 0.174 |
| *P. leniusculus* | 22℃ - 17℃ | 0.156 |
| *P. leniusculus* | 22℃ - 12℃ | 0.996 |
| **Temperature** | **Comparison** | **RIP Value** |
| 12℃ | *P. leptodactylus – P. leniusculus* | 1.72 |
| 17℃ | *P. leptodactylus – P. leniusculus* | 1.82 |
| 22℃ | *P. leptodactylus – P. leniusculus* | 1.77 |

**Figure SI 4:** a) Relative Impact Scores (RIP; based on Functional Response Ratio) and RIPq scores which incorporate the ‘resource reproductive qualifier’ for between-temperature comparisons for *Pontastacus leptodactylus*. The resource reproductive qualifier was based on fecundity index (number of eggs/wet weight) of female *Gammarus fossarum* at different temperatures (Pöckl, 1993). b) Relative Impact Scores (RIP; based on Functional Response Ratio) and RIPq scores for between-temperature comparisons for *P. leptodactylus*. The resource reproductive qualifier was based on average egg number per ovigerous female *Gammarus pulex* at different temperatures (Hynes, 1955). Points above the dashed yellow line indicate greater Relative Impact Potential from the first temperature stated compared to the second e.g. at 17 compared to 9℃.


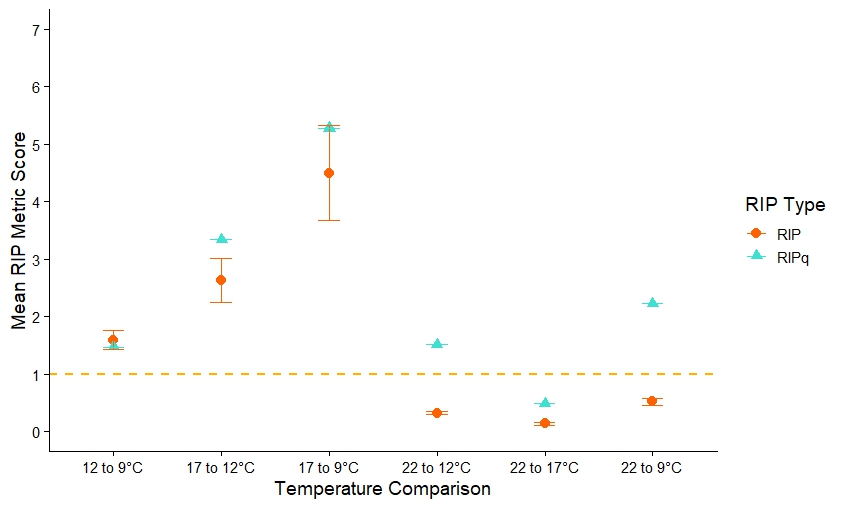


a)


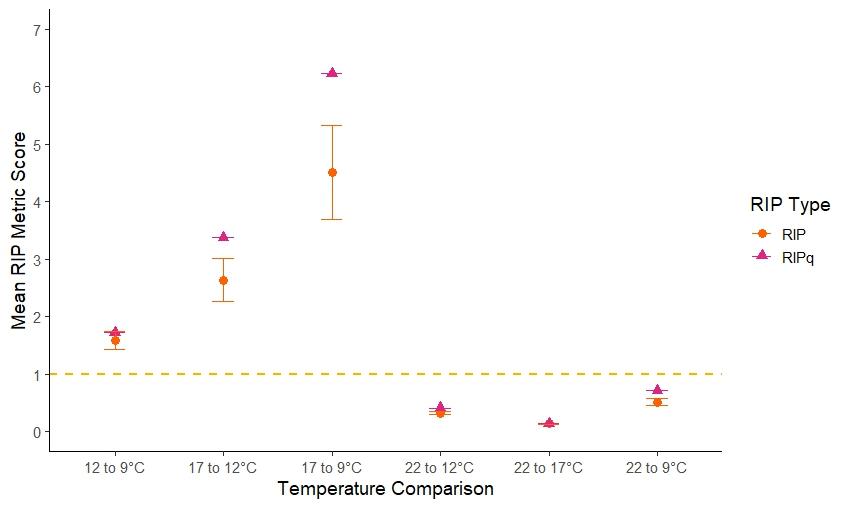


b)

## Supplementary Information 5

## Functional response curves within species. Functional response curves had broadly overlapped confidence intervals for *P. leptodactylus* compared between temperature (Figure SI4). For *P. leniusculus* 12, 17 and 22℃ curves confidence intervals overlapped, as did 9 and 17℃. However, at 9℃ *P. leniusculus* functional response curves diverge from 12 and 22℃ curves, possessing a significantly different curve at low densities (ca. 0 - 18) (Figure SI4 - Left). For *P. leniusculus* at 9℃ the FR curve doesn’t plateau because a high enough density was not used, thus saturation didn’t occur, producing an unreliable and non-significant handling time.


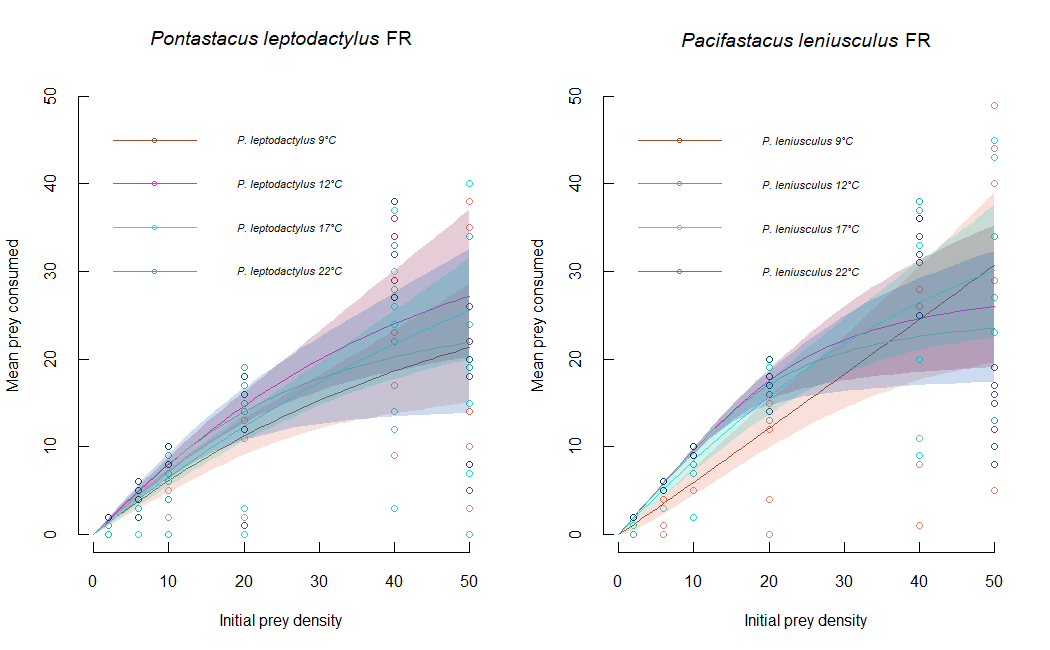


A)

B)

**Figure SI5:** Functional responses curves, with raw mean prey consumed data points, of *Pontastacus leptodactylus* and *Pacifastacus leniusculus* towards *Gammarus pulex* at temperature treatments: 9, 12, 17 and 22 ℃. Shaded areas are 95% bootstrapped CIs, and darker areas represent overlap.

## Supplementary Information 6

Mechanistic comparisons of attack rate and handling times within and between species using z-tests in Frair compare (Juliano 1998).

**Table SI 6**: z-test comparisons to determine significant differences in attack rate or handling time between species at different temperature conditions: 9, 12, 17 and 22℃. 9℃ was not compared for *Pacifastacus leniusculus*.

| **Species Comparison** | **Temperature** | **Estimate** | ***z*** | ***p*-value** |
| --- | --- | --- | --- | --- |
| **Attack rate** |  |  |  |  |
| *P. leniusculus - P. leptodactylus* | 12℃ | 2.95 | 3.69 | <0.001 |
| *P. leniusculus - P. leptodactylus* | 17℃ | 1.065 | 3.72 | <0.001 |
| *P. leniusculus - P. leptodactylus* | 22℃ | 3.00 | 3.06 | <0.01 |
| **Handling time** |  |  |  |  |
| *P. leniusculus - P. leptodactylus* | 12℃ | 0.01 | 2.73 | <0.01 |
| *P. leniusculus - P. leptodactylus* | 17℃ | 0.0041 | 1.020 | 0.30 |
| *P. leniusculus - P. leptodactylus* | 22℃ | 0.0030 | 0.67 | 0.49 |

**Table SI 6.2:** z-test comparisons to determine significant differences in attack rate or handling time within species between temperature conditions: 9, 12, 17 and 22℃. 9℃ was not compared for *Pacifastacus leniusculus*.

| **Species** | **Comparison** | **Estimate** | ***z*** | ***p*-value** |
| --- | --- | --- | --- | --- |
| **Attack rate** |  |  |  |  |
| *P. leptodactylus* | 9℃ - 12℃ | -0.81 | -2.80 | <0.01 |
| *P. leptodactylus* | 9℃ - 17℃ | -0.063 | -0.35 | 0.72 |
| *P. leptodactylus* | 9℃ - 22℃ | -1.17 | -3.06 | <0.01 |
| *P. leptodactylus* | 12℃ - 17℃ | 0.75 | 2.74 | <0.01 |
| *P. leptodactylus* | 12℃ - 22℃ | -0.35 | -0.81 | 0.41 |
| *P. leptodactylus* | 17℃ - 22℃ | -1.10 | -2.99 | <0.01 |
| *P. leniusculus* | 12℃ - 17℃ | 2.63 | -3.28 | <0.01 |
| *P. leniusculus* | 17℃ - 22℃ | -3.04 | -3.19 | <0.01 |
| *P. leniusculus* | 12℃ - 22℃ | -0.40 | 0.34 | 0.73 |
| **Handling time** |  |  |  |  |
| *P. leptodactylus* | 9℃ - 12℃ | 0.00171 | 0.30 | 0.76 |
| *P. leptodactylus* | 9℃ - 17℃ | 0.0081 | 1.43 | 0.15 |
| *P. leptodactylus* | 9℃ - 22℃ | -0.010 | -1.74 | 0.08 |
| *P. leptodactylus* | 12℃ - 17℃ | 0.0063 | 1.42 | 0.15 |
| *P. leptodactylus* | 12℃ - 22℃ | -0.012 | -2.46 | <0.05 |
| *P. leptodactylus* | 17℃ - 22℃ | -0.018 | -3.76 | <0.001 |
| *P. leniusculus* | 12℃ - 17℃ | 0.013 | 3.75 | <0.001 |
| *P. leniusculus* | 17℃ - 22℃ | -0.017 | -4.78 | <0.0001 |
| *P. leniusculus* | 12℃ - 22℃ | -0.0047 | -1.32 | 0.18 |

## Bibliography

Hynes HBN 1955. The Reproductive Cycle of Some British Freshwater Gammaridae. Journal of Animal Ecology. 24: 352–387.

Juliano SA (1998) Nonlinear Curve Fitting: Predation and Functional Response Curves. In: Design and Analysis of Ecological Experiments. Chapman and Hall/CRC

Pöckl, M. (1993) Reproductive potential and lifetime fecundity of freshwater amphipods Gammarus fossarum and G. roeseli in Austrian streams and rivers. Freshwater Biology 30: 73-91.
